# Chronic stability of a neuroprosthesis comprising multiple adjacent Utah arrays in monkeys

**DOI:** 10.1088/1741-2552/ace07e

**Published:** 2023-06-30

**Authors:** Xing Chen, Feng Wang, Roxana Kooijmans, Peter Christiaan Klink, Christian Boehler, Maria Asplund, Pieter Roelf Roelfsema

**Affiliations:** 1 Department of Vision and Cognition, Netherlands Institute for Neuroscience, Meibergdreef 47,1105 BA Amsterdam, The Netherlands; 2 Department of Ophthalmology, University of Pittsburgh School of Medicine, 1622 Locust St, Pittsburgh, PA 15219, United States of America; 3 Experimental Psychology, Helmholtz Institute, Utrecht University, Utrecht, The Netherlands; 4 Laboratory of Visual Brain Therapy, Sorbonne Université, Institut National de la Santé et de la Recherche Médicale, Centre National de la Recherche Scientifique, Institut de la Vision, Paris F-75012, France; 5 Department of Microsystems Engineering (IMTEK), University of Freiburg, Georges-Köhler-Allee 103, 79110 Freiburg, Germany; 6 BrainLinks-BrainTools Center, University of Freiburg, Georges-Köhler-Allee 201, 79110 Freiburg, Germany; 7 Freiburg Institute for Advanced Studies (FRIAS), University of Freiburg, Albertstraße 19, 79104 Freiburg, Germany; 8 Chalmers University of Technology, Chalmersplatsen 4, 412 96 Gothenburg, Sweden; 9 Department of Integrative Neurophysiology, VU University, De Boelelaan 1085, 1081 HV Amsterdam, The Netherlands; 10 Department of Psychiatry, Academic Medical Center, Postbus 22660, 1100 DD Amsterdam, The Netherlands

**Keywords:** non-human primate, V1, V4, blindness, neuroprosthesis, microstimulation, Utah arrays

## Abstract

**Objective:**

Electrical stimulation of visual cortex via a neuroprosthesis induces the perception of dots of light (‘phosphenes’), potentially allowing recognition of simple shapes even after decades of blindness. However, restoration of functional vision requires large numbers of electrodes, and chronic, clinical implantation of intracortical electrodes in the visual cortex has only been achieved using devices of up to 96 channels. We evaluated the efficacy and stability of a 1024-channel neuroprosthesis system in non-human primates (NHPs) over more than 3 years to assess its suitability for long-term vision restoration.

**Approach:**

We implanted 16 microelectrode arrays (Utah arrays) consisting of 8 × 8 electrodes with iridium oxide tips in the primary visual cortex (V1) and visual area 4 (V4) of two sighted macaques. We monitored the animals’ health and measured electrode impedances and neuronal signal quality by calculating signal-to-noise ratios of visually driven neuronal activity, peak-to-peak voltages of the waveforms of action potentials, and the number of channels with high-amplitude signals. We delivered cortical microstimulation and determined the minimum current that could be perceived, monitoring the number of channels that successfully yielded phosphenes. We also examined the influence of the implant on a visual task after 2–3 years of implantation and determined the integrity of the brain tissue with a histological analysis 3–3.5 years post-implantation.

**Main results:**

The monkeys remained healthy throughout the implantation period and the device retained its mechanical integrity and electrical conductivity. However, we observed decreasing signal quality with time, declining numbers of phosphene-evoking electrodes, decreases in electrode impedances, and impaired performance on a visual task at visual field locations corresponding to implanted cortical regions. Current thresholds increased with time in one of the two animals. The histological analysis revealed encapsulation of arrays and cortical degeneration. Scanning electron microscopy on one array revealed degradation of IrO*x* coating and higher impedances for electrodes with broken tips.

**Significance:**

Long-term implantation of a high-channel-count device in NHP visual cortex was accompanied by deformation of cortical tissue and decreased stimulation efficacy and signal quality over time. We conclude that improvements in device biocompatibility and/or refinement of implantation techniques are needed before future clinical use is feasible.

## 1 Introduction

Approximately 40 million people worldwide are blind, leading to difficulties in navigation, reading, and face and emotion recognition, which impair social interaction, reduced workforce participation and significant economic losses [1]. When blindness stems from damage that occurs early along the visual processing pathway but spares part of the retina, therapies that target the retina or the eyeball offer hope for this group of people [2]. However, for those individuals who sustain extensive and irreversible damage to the eye or the optic nerve, vision restoration would require interfacing with the central nervous system [3]. Pioneering work in the 1960s by Button and Putnam, and Brindley *et al* showed that electrical stimulation of the occipital lobe produces visual experiences in blind subjects [4, 5].

It is now well established in both humans [4–19] and animals [20–33] that electrical stimulation of the visual cortex evokes the percept of a dot of light, known as a ‘phosphene’. The location of a phosphene in the visual field is consistent across sessions and depends on the site of stimulation relative to the retinotopic maps in the visual cortex [17, 21, 23, 24, 33–35]. Recently, stimulation of the visual cortex via a 96-channel Utah array allowed a blind volunteer to perceive simple shapes [19] within a small region of the visual field (less than 4 degrees of visual angle (dva)).

However, studies on simulated phosphene vision [36–43] indicate that restoration of functional vision in blind individuals is likely to require at least hundreds to thousands of electrodes, distributed over a large portion of the visual field. This range might be an underestimate given that several of these studies [37–40] assumed that it would be possible to accurately control the perceived luminance of phosphenes in real time, but this has not been realized to date. Furthermore, the visual cortices are highly folded with a complex three-dimensional structure, with only a small part of each visual area at the surface of the brain and accessible to penetrating electrodes, whereas the rest of the cortex is buried within sulci. If a future neuroprosthestic device interfaces with only a small portion of the visual field representation in each visual area, phosphene generation would be limited to small patches of the visual field. Lastly, implant longevity remains a challenge- ideally, a device should remain functional for decades, if not a lifetime. To date, however, clinical studies using microelectrodes have achieved implantation for 4–6 months, followed by device explantation upon conclusion of the study [15,19].

**Figure 1:**
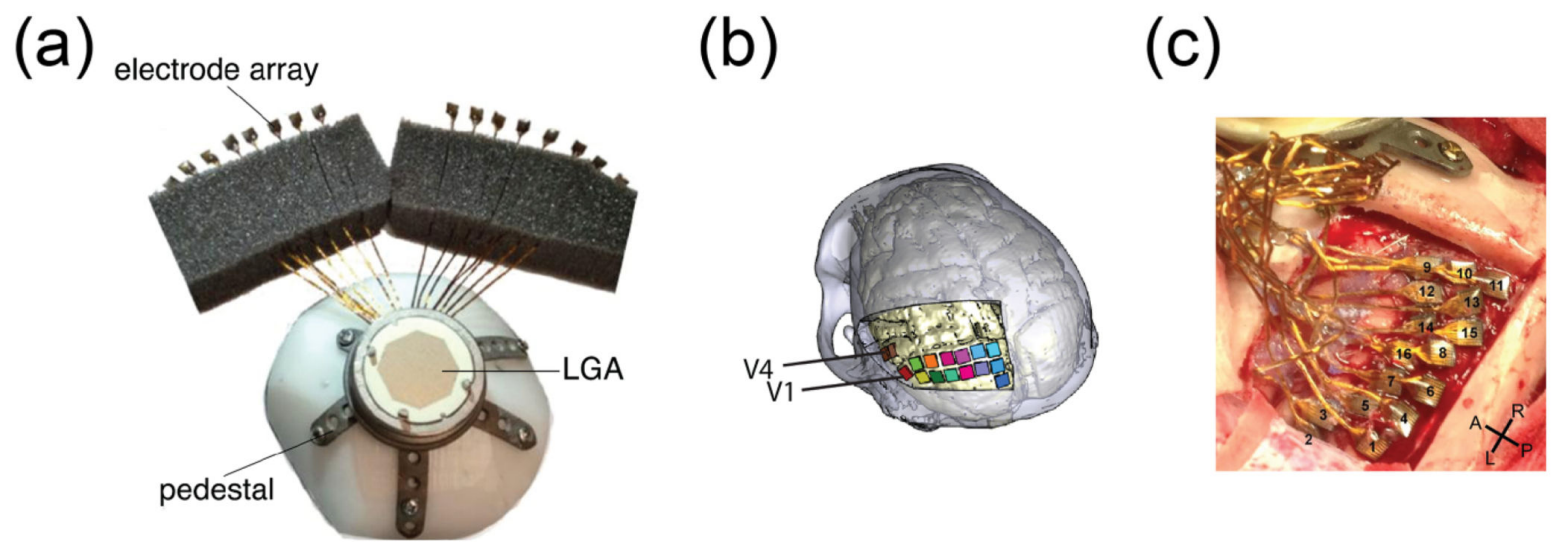
1024-channel neuroprosthesis. (a), Photograph of the implant, consisting of a 1024-channel cranial pedestal connected to 16 Utah arrays. (b), Locations of arrays in areas V1 and V4 of the visual cortex in the left hemisphere of monkey L. (c), Photograph of implanted arrays and wire bundles, taken during surgery in monkey L. Arrays are labelled in black. A: anterior; P: posterior; L: left; R: right.

To test the efficacy of generating phosphenes across a larger region of the visual field and obtaining recognizable shapes, we developed a chronically implantable 1024-channel neuroprosthesis system for recording from and stimulation of the visual cortex in monkeys at high spatial and temporal resolution [33]. The device was used to deliver microstimulation via multiple electrodes simultaneously and thereby generate recognizable shapes such as letters, composed of phosphenes. The implant consisted of 16 Utah electrode arrays (Blackrock Neurotech), attached via 7 cm-long wire bundles to a customized, in-house-designed, 3D-printed pedestal (figure 1(a)). Each array contained an 8-by-8 grid of 64 iridium-oxide electrodes, yielding a total of 1024 electrodes per subject. Fourteen arrays were implanted in V1 and two arrays in V4 (figures 1(b) and (c)).

We used this 1024-channel system for chronic recording and/or stimulation of the cortex, providing a large-scale brain interface that could have a range of future clinical applications, including visual, motor and somatosensory prostheses. Due to the large number of arrays, which were densely tiled across the visual cortex, we considered the possibility that the implant might cause more tissue damage than a single array. Hence, we evaluated the chronic stability and efficacy of the 1024-channel device in two sighted rhesus macaque monkeys over a period of 3–3.5 years. We assessed the longevity of the implant and the health of the animals, and examined the number of channels on which phosphene percepts could be elicited, at different time points after implantation. We also determined the minimum stimulation currents required for phosphene perception. Furthermore, we measured the animals’ performance on a visual task several years after implantation, to examine possible adverse effects on cortical integrity and visual perception. To examine the quality of the implant and recorded signals across time, we measured electrode impedance, the signal-to-noise ratio (SNR) of visually driven neuronal activity, peak-to-peak voltages of the recorded action potentials, and the number of channels with high-amplitude action potentials. After sacrificing the animals, we carried out histological examinations of the implanted region of cortex, evaluating the mechanical and electrical integrity of the explanted prosthesis, and examining possible prosthesis failure modes.

By assessing the long-term stability and functionality of our implant, we explored the potential of using a 1024-microelectrode device for electrical stimulation of the visual cortex, identifying possible biological and technological challenges for multi-year use in patients.

## 2 Methods

### 2.1 Health and behaviour of animals

All experimental surgical procedures complied with the NIH Guide for Care and Use of Laboratory Animals (National Institutes of Health, Bethesda, Maryland), and were approved by the Institutional Animal Care and Use Committee of the Royal Netherlands Academy of Arts and Sciences. We tested the device in two macaque monkeys, L and A. Each animal received two cranial implants (a head post and a pedestal connected to electrode arrays) during separate surgical procedures under general anaesthesia. At the time of head post implantation, the monkeys were 4 and 5 years old, weighing 6.5 and 7.2 kg, respectively. Upon implantation of electrodes in the visual cortex, they were both 7 years old, weighing 11.0 and 12.6 kg, respectively.

The behaviour and general appearance of the monkeys were monitored daily by lab technicians, animal caretakers and research scientists, and if issues arose, they were addressed. We recorded the weight of the animals in a digital logbook on each training day, and veterinarians carried out regular checks on the animals and a thorough annual examination that included a clinical evaluation and several blood tests. The results did not reveal abnormalities.

### 2.2 Surgery for implanting electrode arrays

A 1024-channel device comprising 16 Utah arrays (Blackrock Neurotech) was implanted in the visual cortex of two monkeys, as described previously [33, 44, 45]. Electrode shanks were 1.5 mm in length, with 400 *μ*m inter-shank spacing. Pre-implantation electrode impedances (measured by Blackrock Neurotech prior to lead attachment) ranged from 6 to 12 kΩ. The reference wires protruded from the wire bundles of odd-numbered arrays and each reference wire served as the reference for two arrays, yielding eight reference wires in total. The wire bundles were attached to a customized 3D-printed titanium cranial implant that was anatomically tailored to the individual monkey, ensuring a good fit with the skull [33, 46].

During the surgery, the skin was opened, a craniotomy was made over the visual cortex, and the dura was reflected. We used an inserter wand (Blackrock Neurotech) to insert each array into the cortex, and used a micropipette to apply small amounts (microliters) of Histoacryl tissue glue to the sides of the array to secure it to the cortex, and to secure the wire bundle to the bone. The arrays and wire bundles were connected to each other due to the glue, forming a large implant that spanned approximately 3 cm of cortex. The dura and craniotomy were closed and excess lengths of wire bundles above the skull were covered with dental cement. The surgery lasted 8–10 h, of which 3–4 h were spent inserting the arrays. The animals received close monitoring for signs of pain or discomfort during the recovery period immediately following surgical implantation of arrays. We administered antibiotics and painkillers in accordance with animal welfare protocols.

### 2.3 Health of cranial implant

We inspected the skin surrounding the implant regularly and cleaned it using Prontosan® Wound Irrigation Solution (Braun). In both monkeys, initial regrowth of the skin over the dental cement was followed by gradual retraction of the skin, exposing the dental cement. A gel formulation (Prontosan® Wound Gel, Braun) was topically applied to the wound margin every other day, and surrounding hair was trimmed. If there were signs of local infection of the wound margin, we took bacterial swabs. In monkey L, antibiotic treatment was carried out for one week at 27 months after array implantation, with daily 1-ml intramuscular injections of Duphatroxim.

### 2.4 Software for stimulus presentation and control

We carried out stimulus presentation and control using a combination of custom-written Matlab scripts, and *Tracker* [47], *Psychophysics Toolbox* [48–50] and *Cogent 2000* (RRID:SCR_015672) software packages.

### 2.5 Current thresholds

**Figure 2:**
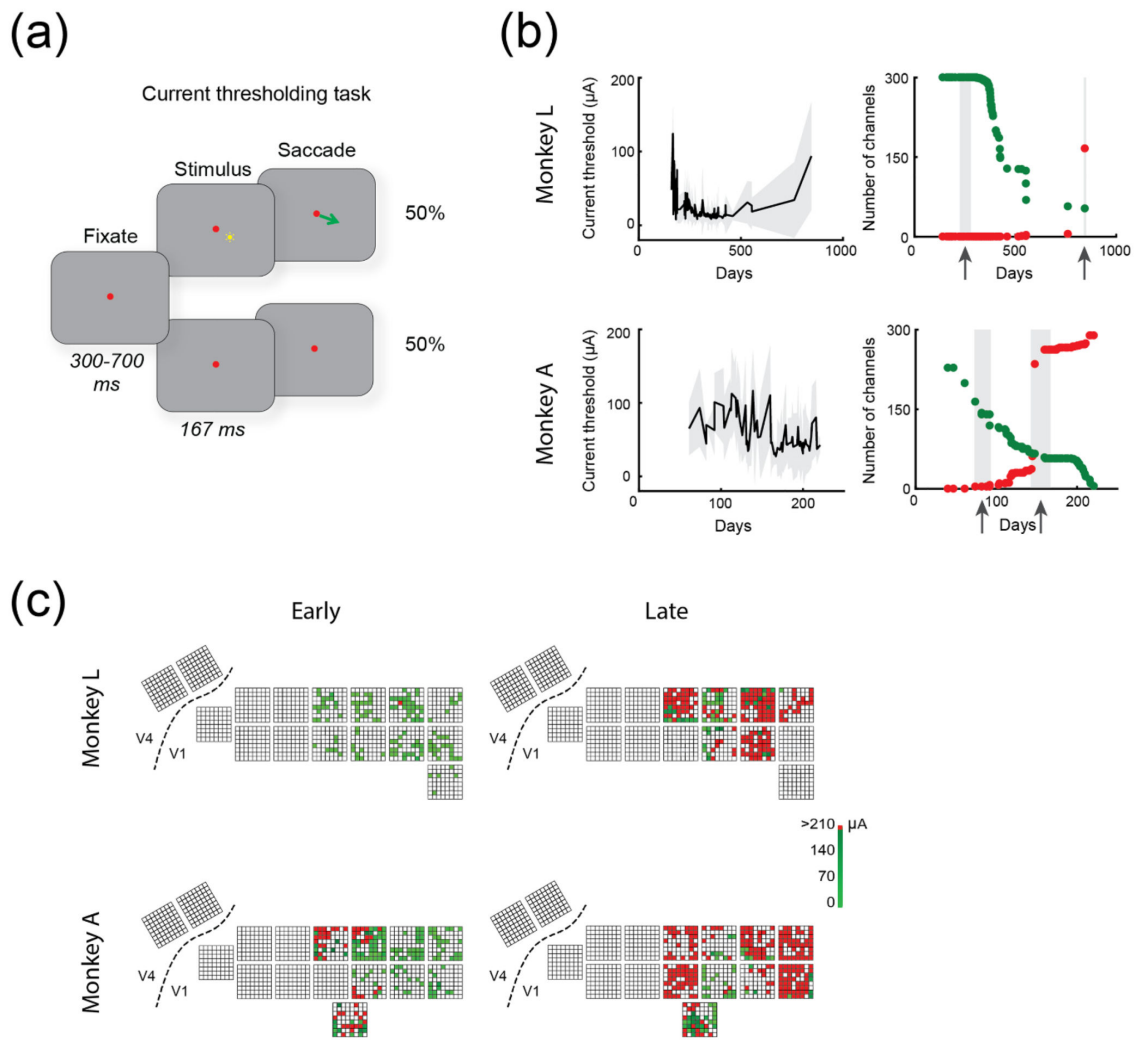
Current thresholding task for phosphene perception. (a), Illustration of the task used to determine the current thresholds. The monkey maintained fixation on a red dot at the centre of the screen. After an interval that ranged from 300 to 700 ms in duration, microstimulation at various current amplitudes was delivered to V1 via a single electrode, and the monkey made a saccade to the phosphene within 250 ms of stimulation onset to obtain a reward. During catch trials, no stimulation was delivered, and the animal was rewarded for maintaining fixation. (b), Left: mean current threshold across channels over time, relative to implantation date. Grey shaded areas show SD. Right: number of channels yielding phosphene perception at this date or later (green); cumulative number of ineffective channels (red). Current thresholds increased significantly with time in monkey L (*t*(26) = −5.1295, *p* < .001). (c), Current thresholds for a subset of electrodes shown at their corresponding location of implantation in the cortex, during an early (left) and late (right) epoch, which have been indicated by grey bars and arrows in panel (b). Green indicates phosphene perception; red indicates no perception. White indicates channels for which current thresholding was not attempted during the respective period. Note that the array positions on the cortex were less orderly than illustrated here (see figure 1(c)).

A visual cortical prosthesis works by delivering electrical currents to the brain tissue to generate phosphene perception. We monitored the number of electrodes that yielded phosphenes, and the current amplitude required for phosphene generation on each electrode. To measure phosphene thresholds, we trained the monkeys on a detection task. Prior to the implantation of electrode arrays, the monkeys performed a saccade task in which they reported the location of a visually presented dot on the screen (figure 2(a)), with an equal proportion of ‘visual trials’ and ‘catch trials’. During visual trials, the animal maintained fixation on a red dot at the centre of the screen, for a period ranging from 300 to 700 ms relative to fixation onset (randomly chosen from a uniform distribution). At the end of this interval, a circular visual target appeared in the bottom-right quadrant of the screen, for a duration ranging from 120 to 150 ms (randomly chosen from a uniform distribution), and a diameter ranging from 0.2 to 0.6 dva. The animal had to make a saccade to the visual target within 250 ms to receive a reward. During catch trials, no visual target was presented, and the animal was rewarded for maintaining fixation throughout the trial. On both visual and catch trials, reward delivery occurred at 1200 ms after fixation onset.

After implantation of the arrays, we replaced presentation of the visual stimulus with delivery of microstimulation on a single electrode. Biphasic cathodic-first monopolar microstimulation was delivered via a train of 50 pulses at 300 Hz, with a pulse width of 170 *μ*s per phase and an inter-phase interval of 60 *μ*s. The animal had to make a saccade to a target window (of 17 dva in diameter that spanned the bottom-right quadrant of the screen) within 250 ms to receive a reward. During catch trials, no microstimulation was delivered, and the animal had to maintain fixation throughout the trial. On both microstimulation and catch trials, reward delivery occurred at 1200 ms after fixation onset. Performance during microstimulation trials was calculated as the number of hits divided by the sum of hits and misses, while performance during catch trials was calculated as the number of correct rejections divided by the sum of false alarms and correct rejections.

The current amplitudes used for microstimulation were drawn from a fixed range of values on a logarithmic scale, from 1 to 210 *μ*A. This logarithmic distribution ensured that accurate current threshold values could be obtained for all channels, including those with very low current thresholds, where even small changes in current amplitude (on the order of a few *μ*A) influenced perception, as predicted by Weber’s law [51].

A staircase procedure was used to determine the current threshold for each electrode (described in detail in [33]). Briefly, the monkey performed the saccade-to-phosphene task, in which microstimulation was delivered on 50% of trials. The staircase procedure was terminated after 10–20 microstimulation trials were obtained. We plotted the proportion of hits and misses against current amplitude, and fit the data with a Weibull function, yielding a psychometric function for phosphene perception as function of current amplitude. The current threshold for a given channel was defined as the current amplitude that yielded an equal proportion of hits and misses. The threshold value was stored and used to set the initial current amplitude value during subsequent current thresholding sessions for that channel.

To identify changes in current thresholds with time, we identified sessions at early and late time points, during which current thresholding was carried out for many electrodes. We selected channels for which a current threshold was obtained during both early and late periods (pooled across sessions for each period) and performed a paired-sample *t*-test using the earliest and last current thresholds obtained for these channels. Note that surgical implantation of the arrays was followed by a recovery period of one month, after which the monkeys were engaged in other (unrelated) tasks that did not involve stimulation. Hence, current thresholding began at around 3 and 2 months after the surgery in monkeys L and monkey A, respectively. Across channels, current thresholding was carried out on a mean of 10.5 ± 6.8 (SD) and 4.5 ± 6.0 sessions for monkeys L and A, respectively.

### 2.6 Visual detection task

**Figure 3:**
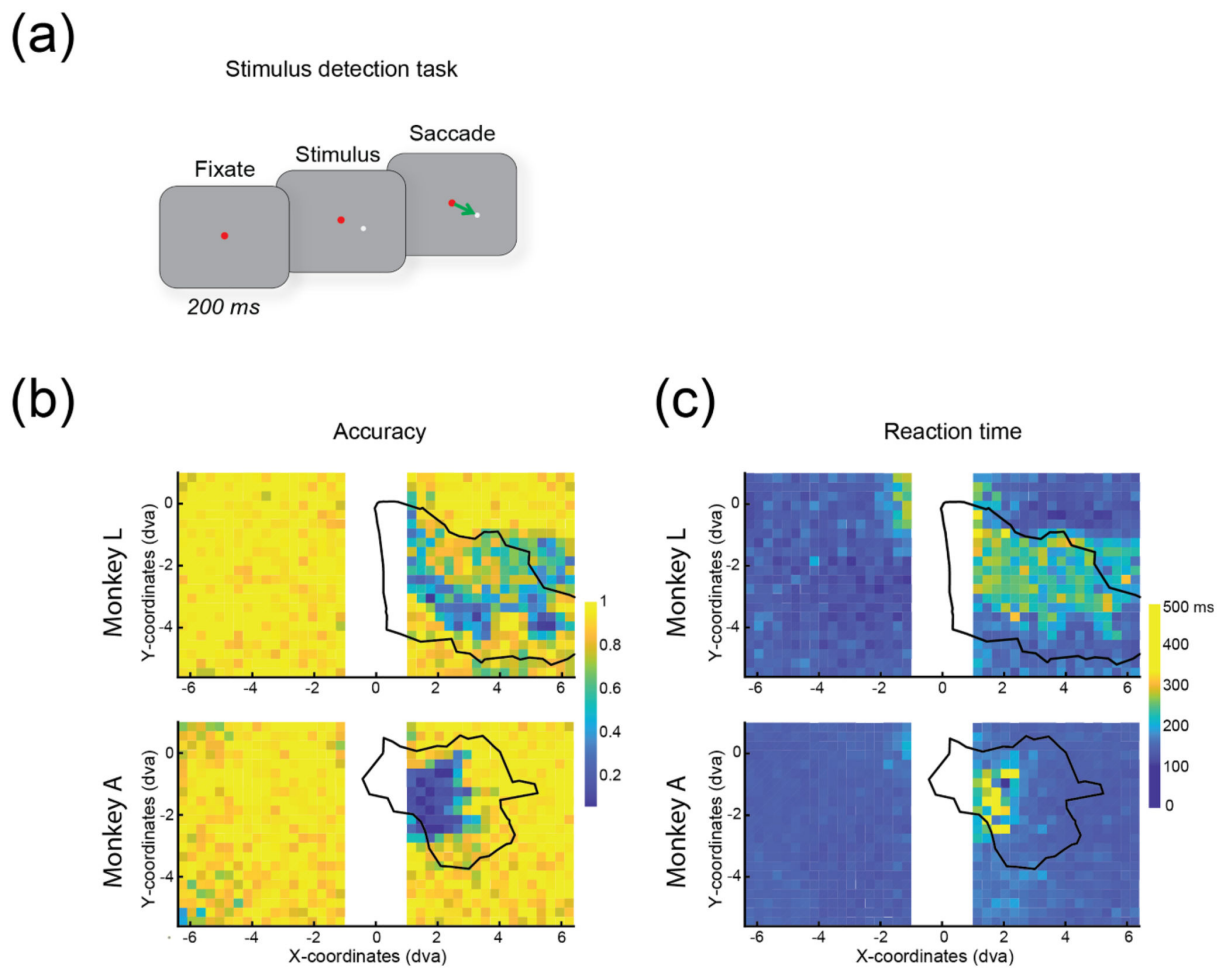
Performance on the visual detection task. (a), Illustration of the task. The monkey initiated the trial by fixating on a red dot at the centre of the screen. After 200 ms, a small grey circle stimulus was presented and the monkey was rewarded for making a saccade to the stimulus within 200 ms of its onset. (b) and (c), Performance on the visual detection task, showing accuracy (b) and reaction time (c) at each stimulus location. The black line demarcates the region of the visual field corresponding to the retinotopy of the implanted cortex. Significant decreases in accuracy (monkey L: *t*(217) = 23.17, *p* < .001; monkey A: *t*(134) = 14.20, *p* < .001) and increases in reaction time (monkey L: *t*(217) = −20.07, *p* < .001; monkey A: *t*(134) = −10.61, *p* < .001) were observed.

We trained the monkeys to perform a stimulus detection task (figure 3(a)), to assess vision at various locations throughout the visual field. They initiated fixation at a central spot, and after 200 ms a light grey (30.3 cd m^−2^) circle stimulus was presented on a grey background with a luminance of 16.8 cd m^−2^. The monkeys made a saccade to a target window of 2 dva in diameter centred on the stimulus within 200 ms of stimulus onset, to receive a liquid reward. In the initial stages of training, the stimulus was large and appeared at the centre of the screen, overlapped by the fixation spot. Once task performance was high, the stimulus was moved away from the centre of the screen and its diameter was reduced. In the final version of the task, the stimulus had a diameter of 0.2 dva and was presented at different locations in the lower visual field, following a grid layout (38 by 23 locations on the *x*- and *y*-a*x*is, respectively, with 0.3 dva spacing between adjacent grid points). Task performance was calculated across a mean of 14 trials per condition (*μ_N_* = 14.3, SD = 2.7 in both monkeys, across 12 and 19 sessions in monkeys L and A respectively), yielding a map of detection performance in the lower visual field.

The arrays were implanted in the left hemisphere and the V1 receptive fields were located in the lower right hemifield, spanning the central 8 and 5 degrees of the visual field in monkeys L and A, respectively. Hence, we hypothesized that tissue damage would cause visual deficits in the lower right hemifield. To determine the region of the visual field corresponding to implanted cortical regions, we determined receptive field (RF) locations for each channel using an RF mapping task [45], and identified the boundary of the ‘conglomerate RFs’ for each monkey. First, we selected channels with SNR of >2, excluding channels with outlying RF centres and sizes (>3 SDs from the mean). We drew a line bounding the RF centres of these channels, yielding the boundary of the ‘conglomerate RFs’. For each stimulus location in the visual acuity task, we determined whether the stimulus lay within or outside this boundary. Mean task accuracy and reaction time were calculated across trials for each stimulus location and compared between within-conglomerate-RF and outside-conglomerate-RF conditions.

### 2.7 Visually evoked activity and signal-to-noise ratios (SNRs)

**Figure 4:**
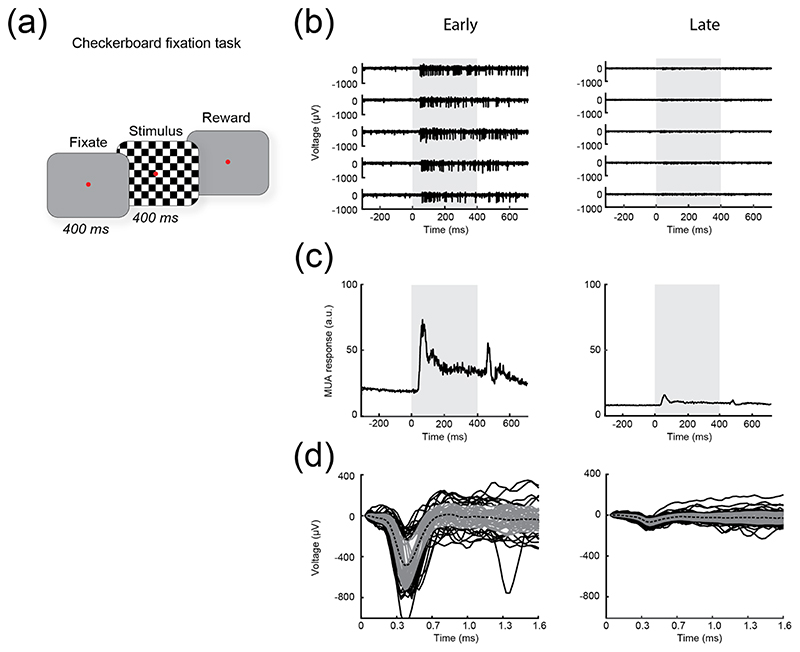
Checkerboard stimulus used to assess signal quality. (a), Illustration of the task. The monkey initiated a trial by fixating on a red dot at the centre of the screen. After 400 ms, a full-screen checkerboard stimulus was presented for 400 ms, and the monkey was rewarded for maintaining fixation. (B)–(D), Data from an example channel (channel 40 on array 11, marked by red circles in figures 5(a) and (c)) during early and late sessions (91 and 279 d post-implantation, respectively). (b), Raw data (band-pass filtered from 500 to 9000 Hz) on 5 example trials, showing visually evoked responses. Grey: stimulus presentation from 0 to 400 ms. (c), Mean visually evoked response across trials, used to calculate the SNR for each session (corresponding to data marked by red circles in figures 5(a) and (c)). Grey: stimulus presentation from 0 to 400 ms. (d), Snippets used to calculate peak-to-peak voltage of action potentials, and mean waveform and SD (black dotted line and grey shaded areas) across all snippets from the session.

We assessed the quality of neuronal signals on each channel using visually evoked responses to a full-screen checkerboard stimulus that was displayed for 400 ms, while the monkey maintained fixation on a dot at the centre of the screen (figure 4(a)). The checkerboard squares were 1 dva in diameter, and the luminance of the black and white squares was 0 and 92.1 cd m^−2^, respectively. Prior to stimulus onset, the screen was grey (with a luminance of 14.8 cd m^−2^).

The task was carried out across 21 and 8 recording sessions, distributed over a 3.5 year and 3 year period in monkeys L and A, respectively. The raw neuronal signal (sampling frequency of 30 kHz) on each of the 1024 channels was processed to obtain envelope multiunit activity (MUAe [52], down-sampled to 1 kHz). The SNR on each channel was calculated as follows: the spontaneous activity level was calculated as the mean activity in the 300 ms time window prior to stimulus onset, while the noise level was calculated as the standard deviation of the activity in the same window across trials. Next, the MUAe data was smoothed with a moving average of 20 bins (corresponding to 20 ms), and we identified the peak response elicited by the stimulus. The mean spontaneous activity level was subtracted from this peak activity level, yielding the response level relative to baseline, and the result was divided by the standard deviation of the baseline activity, yielding the SNR (see equation (1)):

(1) $$\text{SNR}=\frac{{\text{Peak}}_{\text{stimulus}-\text{evoked}}-{\text{Mean}}_{\text{spontaneous}}}{{\text{SD}}_{\text{spontaneous}}}.$$

Supplementary figure 1 shows the mean responses elicited by the checkerboard stimuli across all 1024 channels, for example early (left) and late (right) sessions (days 75 and 727 for monkey L; days 33 and 384 for monkey A). For each channel, the range on the *Y*-axis is set to be equal between early and late sessions. Across channels, the mean and SD of the range on the *y*-axis are 5.83 ± 5.79, in arbitrary units (a.u.). figure 4 shows raw data (figure 4(b)) and MUAe (figure 4(c)) from an example channel (channel 40 on array 11) during early and late sessions (91 and 279 d post-implantation, respectively). We examined the SNR values across all channels and sessions (supplementary figure 2(a)), as well as the physical locations of the channels and arrays on the cortex.

To compare SNR values between the period immediately following array implantation to those obtained at a later point in time, we combined data across the first two sessions (categorized as ‘early sessions’) and across the last two sessions (‘late sessions’) and compared SNR values for all 1024 channels. A one-way analysis of variance (ANOVA) was carried out, with time as a factor (‘early’ or ‘late’). Violin plots were generated to visualize changes in SNR with time, using open-source Matlab scripts [53]. To examine changes in the number of channels with good signal quality, we calculated the number of channels with SNR >1 for each session and carried out a linear regression.

### 2.8 Signal amplitude and peak-to-peak voltage

The dataset obtained for the calculation of SNR was also used to calculate peak-to-peak voltage during visual stimulus presentation and to identify channels with high-amplitude signals, providing two additional measures of signal quality [54]. We used automated techniques [54, 55] instead of manual spike sorting to avoid bias when assessing the quality of spike recordings [56].

The methods and values used are identical to those described by Hughes *et al* [54]. For each trial and electrode, we calculated the root-mean-square level of baseline activity. If the voltage signal during stimulus presentation crossed a negative threshold of −4.5 times the root-mean-square level, the time of threshold crossing was noted and a 1.6-ms snippet of signal was stored (starting ten samples before threshold crossing). Snippets from an example channel (channel 40 on array 11) during early and late sessions (91 and 279 d post-implantation, respectively) are shown in figure 4(d).

For each electrode, we identified the largest 2% of snippets across trials, and calculated the average waveform across these snippets. We then measured the peak-to-peak voltage of the average waveform. Electrodes with a trial-wise snippet occurrence rate of >1.67 Hz and a peak-to-peak voltage of >30 *μ*V in the average waveform were used for further analysis. If the peak-to-peak voltage on a given electrode was >100 *μ*V, it was deemed to yield high-amplitude spikes. Violin plots were generated to visualize changes in peak-to-peak voltage with time, using open-source Matlab scripts [53]. We calculated the mean peak-to-peak voltage across channels for each session, and carried out a linear regression against the number of days post-implantation. If the slope of the regression line was significantly different from 0, this indicated a change in mean peak-to-peak voltage over time. We examined the peak-to-peak voltages across all channels and sessions (supplementary figure 2(b)), and the locations of the channels and arrays on the cortex.

### 2.9 Impedance values

We measured electrode impedance at 1 kHz at multiple time points during the implantation period, across 19 and 9 sessions in monkeys L and A, respectively, using the Impedance Tester function in the Central Software Suite (Blackrock Neurotech). Following perfusion of the animals and explantation of the arrays, we measured electrode impedance at 1 kHz using a nanoZ (White Matter LLC, Seattle, US). We examined impedances across all channels and sessions (supplementary figure 2(c)), and the locations of the channels and arrays on the cortex.

### 2.10 MRI template registration for histological analysis

In preparation for the histological analysis, a 3D rendering of the magnetic resonance imaging (MRI) template of the rhesus macaque brain, compiled across 31 macaques (NIMH Macaque Template, version 2; NMTv2) was non-linearly co-registered to the anatomical T1-weighted scan of the brain of monkey L, obtained before implantation of electrode arrays. First, a T1-weighted MRI (3D-FFE; GR-MP, TE = 7 ms, TR = 15 ms, flip angle = 8°, 0.5 × 0.5 × 0.5 mm isotropic voxels) was acquired on a 3 T Philips Ingenia MR scanner using a 32-channel head coil while the animal was anesthetized with an intramuscular injection of Medetomidine (0.08 ml kg^−1^) together with Ketamine (0.07 ml kg^−^ 1). Digital Imaging and Communications in Medicine (DICOM) images were converted to *nifti* files using *dcm2niix* [57] and the individual anatomical scan was nonlin-early registered to the NMTv2 template using AFNI’s *@animal_warp*er function [58]. This yielded an anatomical model of the intact brain (pre-implantation), providing a basis of comparison with the implanted, post-mortem brain.

### 2.11 Perfusion and tissue processing

Euthanasia was carried out at 3 and 3.5 years of implantation in monkeys L and A, respectively. We perfused monkey A transcardially with a 4% solution of phosphate-buffered paraformaldehyde. Due to an error during the perfusion procedure, the brain of monkey L was incompletely fixed, hence we fixed it by immersion.

We detached the meninges from the skull and observed a mass of newly formed tissue that encapsulated the implants. We observed that on some arrays, several electrode tips remained visible and protruded from the encapsulating tissue. We identified the channels that remained exposed and the arrays to which they belonged. The encapsulation tissue was carefully dissected away, allowing retrieval and storage of arrays, wire bundles, and reference wires. In monkey L, to avoid damaging the brain tissue, we cut the wire bundles connecting the arrays to the pedestal. In monkey A, we observed that the wire bundles on four of the most laterally located arrays (arrays 1, 2, 3 and 4) were severed. Furthermore, the electrodes were detached from their base on array 6, and the wire bundle of array 12 was severed during dissection. The remaining ten arrays remained connected to the pedestal.

In both monkeys, the fixed brain and explanted 1024-channel implant were stored in 30% sucrose in phosphate-buffered saline. We observed deformations of the cortical surface of the implanted hemisphere due to the presence of the implant and encapsulating tissue, and created a digital model of the cortex prior to slicing of the brain for histology. We first produced a negative mould of the cortex in 2% agar, followed by a positive mould in silicone (OOMOO™, 25 Shore). We scanned the silicone model using a desktop 3D scanner (EinScan-SP, SHINING 3D) at a resolution of 50 *μ*m, yielding a digital copy of the cortex, and post-processed it to yield a clean model.

We separated the two hemispheres and dissected two blocks, posterior to the lunate sulcus. We froze the blocks by submersion in isopentane cooled to −50 °C, and then cut them into 50 *μ*m-thick sections in the frontal plane, using a cryo-microtome. We stained sections from the two hemispheres (implanted and control) using fluorescent Nissl (1:500; NeuroTrace™ 530/615 Red Fluorescent Nissl Stain, Invitrogen™). We imaged the fluorescent Nissl using a Leica SP8 Confocal microscope (10 × magnification) to evaluate the extent of changes observed in the cortical surface during gross sectioning.

### 2.12 Post-mortem impedances and scanning electron microscopy

In monkey L, as the explanted arrays were detached from the pedestal, one array was selected for closer examination. We chose array 8 as it was in the middle of the implanted arrays, and several of its electrodes (14/64) had been used for stimulation. We carried out sputter coating with 7 nm of Au (CCu-010, Safematic) and obtained scanning electron microscopy images (Helios 5, Thermo Fisher Scientific) at 1250× magnification, for 62/64 electrodes. Two of the electrode shafts were detached from the array and remained in the fibrotic tissue, hence we excluded them from imaging. For each electrode, we coded the silicon tip as intact or broken, and noted any pitting of the silicon surface. We examined SNR and impedance values obtained during the final *in-vivo* measurement (1299 d after implantation) for possible differences between intact and broken electrodes. We compared the identities of electrically stimulated electrodes with those that had visible pitting of the Si surface.

In monkey A, 10 of the 16 explanted arrays (arrays 5, 7–11, 13–16) remained connected to the pedestal via their wire bundles. The arrays were cleaned via immersion in contact lens solution for several days (Etos BV, Netherlands) followed by de-ionized water. The arrays were allowed to dry out after explantation. To test electrical conductance of the explanted device, the legs of the explanted pedestal and the intact Utah arrays were placed in a beaker of saline solution. The contact points on the land grid array of the pedestal were connected to an electronic interface board, providing a passive electrical connection to 32-channel Omnetics connectors. We used a nanoZ device to obtain impedance measurements from the ten connected arrays at 1 kHz. One of the channels on the nanoZ device was not functional, and measurements on that channel were discarded. Measurements from channels with high impedance values (>5000 kOhms, *N =* 4 channels) or that yielded clipped signals due to amplifier saturation (*N* = 111 channels) were also discarded.

## 3 Results

Our previous study demonstrated that a 1024-channel neuroprosthesis reliably induced discriminable percepts consisting of phosphenes in monkeys [33]. Here, we evaluated the effects of long-term implantation on the animals’ health, behaviour, phosphene perception, and visual perception, to better understand the challenges associated with chronic implantation in human patients. We assessed the efficacy of the hardware, the mechanical stability and integrity of the implant, and the quality of recorded neuronal signals. After the conclusion of behavioural experiments, we examined the effects of chronic implantation on the cortical tissue including the degree of tissue damage and gliosis induced by the device. The datasets used in this paper are summarized in table 1.

### 3.1 Health and behaviour

The two monkeys were healthy throughout the implantation period. On all counts, their behaviour and appearance were normal throughout the course of the study. Their weight was monitored regularly and fell within the expected range for the age of the animals. The implant was well attached to the skull for the duration of the experiments and occasional signs of local skin infection at the margin between the skin and the implant were treated (see methods).

### 3.2 Phosphene induction

Previous studies showed that stimulation of the visual cortex via multiple electrodes generates phosphenes that form discriminable percepts [59], such as lines and letters [19, 33, 35, 60]. Here, we examined the number of electrodes that successfully evoked phosphenes over time, to determine the efficacy and longevity of the prosthesis and evaluate the potential of using this technology for a future clinical device.

We first determined the minimal current that gave rise to the perception of phosphenes, on individual electrodes, using a current thresholding task. The monkeys initiated a trial by fixating on a dot at the centre of the screen. On 50% of the trials, after a variable interval (ranging from 300 to 700 ms), we delivered microstimulation to V1 via a single electrode, and the monkey made a saccade to the phosphene to obtain a reward. The other 50% of trials were catch trials in which no stimulation was delivered, and the animal simply maintained fixation.

We varied the current amplitude to determine current thresholds on a subset of channels across the implantation period. To do so, we identified electrodes with impedances of <150 and <300 kOhms, in monkeys L and A, respectively (the cut-off impedance was set at a higher value for monkey A than for monkey L due to the higher impedance levels in this monkey). Current thresholding was carried out for a total of 300 and 372 channels across 102 and 69 sessions in monkeys L and A, respectively (figure 2(b), left).

To evaluate the number of electrodes that successfully elicited phosphenes, we pooled sessions within an earlier and a later period (monkey L, early period: days 229–246, *N =* 137 electrodes, late period: day 845, *N =* 219 electrodes; monkey A, early: 47–64, *N =* 198 electrodes, late: 144–166, *N =* 280 electrodes). These periods were 20 months apart in monkey L and only 3 months apart in monkey A, due to differences between the monkeys in the time required to carry out other experiments. We tallied the number of electrodes that either yielded measurable thresholds or failed to elicit phosphenes.

Note that the main purpose of the current thresholding task was to carry out phosphene detection and phosphene shape discrimination tasks, described in a separate study [33]. Electrodes were selected for stimulation such that the receptive fields of the stimulated neurons collectively formed a shape, such as a line or a letter. Hence, we did not carry out current thresholding across all the electrodes periodically, could not determine the precise date on which stimulation failure occurred on a given channel, and do not have an overview of all effective and ineffective electrodes.

We found that for monkey L, the number of electrodes that were tested and effective decreased from 98% (134/137) to 24% (53/219). Similarly, for monkey A, the number of effective electrodes decreased from 93% (185/198) to 5% (13/280) (figure 2(b), right). The cortical locations of the electrodes that had been tested and their efficacy in generating phosphenes are illustrated in figure 2(c).

We also compared current thresholds between early and late periods, for channels that had been tested and produced phosphenes in both periods. The loss of functional electrodes was accompanied by a significant increase in current thresholds in monkey L, but not in monkey A (monkey L: *μ*_early_ = 19 ± 17 (SD) *μ*A; *μ*_late_= 80 ± 71 *μ*A, *t*(26) = -5.1295, *p* < .001; monkey A: *μ*_ear_ly = 65 ± 45 *μ*A; *μ*_late_= 60 ± 58 *μ*A, *t*(11) = 0.361, *p =* .725, paired-sample *t* -test).

We hypothesized that the decreases in the number of effective channels may have been due to biological factors, such as tissue encapsulation of the electrodes, or to mechanical factors, such as degradation of the electrodes or connector. We therefore carried out a behavioural task to assess the monkeys’ level of vision throughout their visual field, and assessed indicators of signal quality, including SNR and peak-to-peak voltage, to identify potential changes in the recordings and the time course of these changes. Finally, we examined the histology of the visual cortex and the electromechanical integrity of the explanted prosthesis.

### 3.3 Visual detection task

Two to three years after implantation, we trained the monkeys to report the luminance of a visual stimulus and noticed relatively poor performance in the visual field region in which electrodes had been implanted. We therefore suspected that there was damage to the visual cortex. To examine the extent of loss of visual function, we trained the animals on a visual detection task in which they had to report the presence or absence of a visual stimulus. The monkeys started a trial by directing their gaze to a fixation spot at the centre of the screen. They had to make an eye movement to a small (0.2 dva diameter) visual stimulus (figure 3(a)) that was presented at one of several locations on a grid across the lower visual field (38 by 23 locations, see methods). We identified visual field locations corresponding to implanted versus non-implanted regions of cortex, defining ‘conglomerate RFs’ as the region of the visual field that corresponded to the collective RF locations across the electrodes (black outlines in figures 3(b) and (c)). We classified each stimulus as being positioned either inside or outside the conglomerate RFs.

We tested monkey L three years after array implantation. While task accuracy was 92.6 ± 0.5% (mean ± SEM) for stimuli outside the conglomerate RFs, it dropped to 65.2 ± 1.6% for stimuli inside (*t*(217) = 23, *p* < .001) (figure 3(b)). Similarly, in monkey A (tested two years after array implantation), task accuracy outside the conglomerate RFs was 90.1 ± 0.6% but 63.7 ± 3.2% inside (*t*(134) = 14.20, *p* < .001, two-sample *t*-test). These decreases in accuracy were accompanied by increased reaction times (figure 3(c)). The mean RT in monkey L was 151.6 ± 1.3 ms at control locations and 210.1 ± 3.4 ms at conglomerate RF locations (*t*(217) = − 20.07, *p* < .001). In monkey A, it was 153.1 ± 1.2 ms at control locations and 200.1 ± 8.2 ms at RF locations (*t*(134) = −10.61, *p* < .001, two-sample *t*-test). Hence, after several years, the implant caused significant impairments in accuracy in the visual task and increased reaction times, at the V1 visual field representation corresponding to array implantation.

### 3.4 Signal-to-noise ratio (SNR) of the visually driven response

To evaluate the quality of the electrode–tissue interface, we measured visually evoked responses of neurons recorded via the electrodes (figure 4). On each trial, we presented a high-contrast checkerboard stimulus to the monkeys and measured the SNR (defined in the methods) of visually driven neuronal responses in area V1. A high SNR is indicative of a functional electrode that delivers good neuronal signals, whereas a low SNR may indicate electrode failure, connection failure, poor contact between the electrode and the neuronal tissue, and/or tissue gliosis that increases the distance between the electrode and the surrounding neurons.

**Figure 5:**
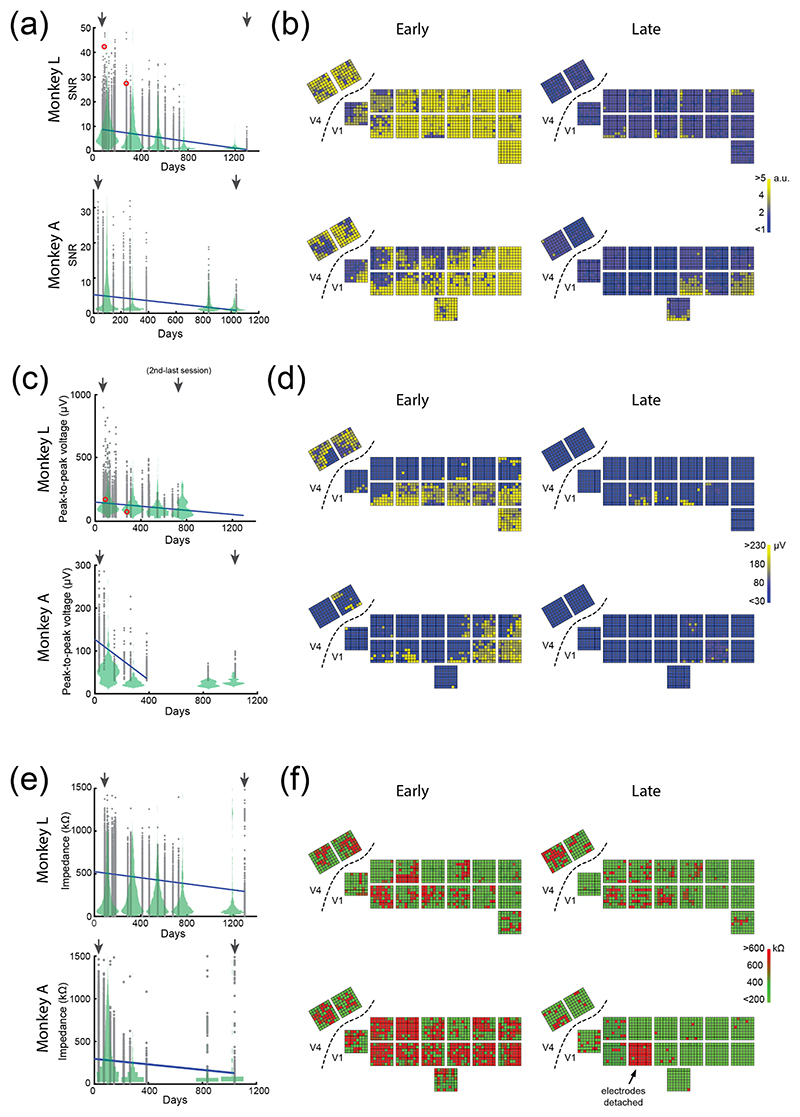
Changes in signal quality and electrode impedance with time. (a), SNR of the visually driven response elicited by a checkerboard stimulus in monkey L (upper row) and monkey A (lower row) relative to implantation date. Violin plots show data averaged across 6 time-bins. Blue line: best-fit line using linear regression. Red circles indicate the example channel shown in figures 4(b)–(d). SNR decreased significantly with time (monkey L: *F*(1,4094) = 1504, *p* < .001; monkey A: *F*(1,4094) = 1327, *p* < .001). (b), SNR values across all arrays during an example early (left) and late (right) session (indicated by the arrows in (a)). (c), Peak-to-peak voltages of action potentials relative to the implantation date. Red circles indicate the example channel shown in figures 4(b)–(d). The number of high-amplitude channels decreased significantly with time (monkey L: *χ*(1,1024) = 369.5, *p* < .001; monkey (a): *χ*(1,1024) = 172.4, *p* < .001). (d), Peak-to-peak voltage at each electrode during example early and late sessions. (e), Impedance values as a function of time after the implantation, for electrodes with an impedance of <2000 kOhms, measured at 1 kHz. Impedance on this subset of electrodes decreased significantly with time (monkey L: *F*(1,4009) = 36.6, *p* < .001; monkey A: *F* (1,4094) = 2633, *p* < .001). (f): Impedance values during example early and late sessions.

We plotted SNR values of all functional electrodes as a function of days after the surgery (figure 5(a)) and examined the spatial distribution of SNR values across all the arrays during example early and late sessions (figure 5(b)). In the period following implantation, a large proportion of channels showed high SNR values of >1 (1019/1024 and 979/1024 channels, at 75 and 69 d after surgery in monkeys L and A, respectively), indicating good signal quality (yellow in figure 5(b)). However, in both monkeys, SNR decreased over time (blue in figure 5(b)). For the statistical tests, we combined the data across the first two sessions and the last two sessions, which were 22 months apart for monkey L and 12 months for monkey A. SNRs decreased significantly in both animals (monkey L: *F*(1,4094) = 1504, *p* < .001; monkey A: *F*(1,4094) = 1327, *p* < .001, one-way ANOVA). The number of electrodes with SNR >1 also decreased over time (supplementary figure 2(a), monkey L: *t*(19) = − 5.223, *p* < .001, monkey A: *t*(6) = − 2.833, *p =* .0299, linear regression).

In summary, the decrease in phosphene perception was accompanied by a decrease in SNR over the years. The deterioration occurred more gradually in monkey L than in monkey A.

### 3.5 Peak-to-peak voltage of detected action potentials

As another measure of recording quality, we examined the amplitude of the recorded action potentials (in *μ*V) [54]. The mean peak-to-peak voltage of detected action potentials decreased significantly with time in monkey L (*t*(18) = −4.694, *p* < .001). In monkey A, we observed a sharp drop during the first few months, after which it stabilized at a low value (during the first six sessions, distributed over 13 months of implantation: *t*(4) = − 3.0197, *p =* .0392; all sessions: *t*(4) = *—*2.678, *p =* .0553, linear regression, figures 5(c) and (d)). We also examined the number of high-amplitude channels (>100 *μ*V peak-to-peak voltage) per session, and found that they decreased significantly with time in both monkeys, from 313/1024 channels at day 75, to 0 channels at day 1299 in monkey L (*χ*(1,1024) = 369.5, *p* < .001, Chi-square test) and from 159 channels at day 33, to 0 at day 145 in monkey A (*χ*(1,1024) = 172.4, *p* < .001, supplementary figure 2(b)).

### 3.6 Impedance values throughout implantation

Electrode impedance depends on numerous factors, including the health and proximity of biological tissue to the electrode, electrode and implant materials, and the physical integrity of the electrode and its connections. A sudden increase in impedance may indicate that an electrical connection has been severed, while gradual changes may point to fibrous encapsulation of the implant. However, the relationship between impedance and recording quality is complex and remains only partially understood [61]. Previous studies reported that gradual declines in neuronal signal quality are accompanied by decreases in impedance [62], and also that encapsulation of electrodes can lead to lower impedances [63]. Hence, impedance is often measured prior to and during implantation, providing an indicator of electrode integrity and the stability of the electrode–tissue interface [64].

The impedance values followed a bimodal distribution in both monkeys. The majority of channels (97.9% in monkey L and 99.4% in monkey A, at day 88 and 33, respectively) had an impedance of <2000 kOhms. However, others had an impedance of >3000 kOhms (supplementary figure 2(c), bottom) and we assumed that these channels had non-functional electrodes or broken connections. The number of channels with an impedance larger than 3000 kOhms increased over time (monkey L: 22/1024 channels on day 88 and 98/1024 channels on day 727; monkey A: 6/1024 channels on day 33 and 34/1024 channels on day 386). However, the identity of these high-impedance channels changed, with only 5 and 3 channels in common between early and late sessions in monkeys L and A, respectively. These high-impedance channels were excluded from subsequent analysis [23].

Next, we examined the channels with impedances of <2000 kOhms and observed a general decrease in impedance values in both animals, with a particularly rapid decline in monkey A (figures 5(e) and (f)). To quantify these observations, we combined data across the first and last two measurement sessions, which were 21 months apart in monkey L and 12 months apart in monkey A. In both animals, we observed a significant decrease in impedance with time (one-way ANOVA with time as factor; monkey L: *F*(1,4009) = 36.6, *p* < .001; monkey A: *F*(1,4094) = 2633, *p* < .001). These results, taken together, suggest that there were two categories of channels, a higher-impedance group that may correspond to non-functional electrodes, and a lower-impedance group with electrodes that remained partly functional but for which impedance decreased over time.

### 3.7 Tissue dissection and implant integrity

We sacrificed the monkeys 3–3.5 years after implantation. In both monkeys, two of the legs of the titanium pedestal were partially covered by bone, indicating good integration between the pedestal and skull. The skull underlying and adjacent to the dental cement flanking the transcranial wire bundles was replaced with softer, whitish tissue spanning approximately 26 and 19 mm in diameter in monkeys L and A, respectively.

**Figure 6:**
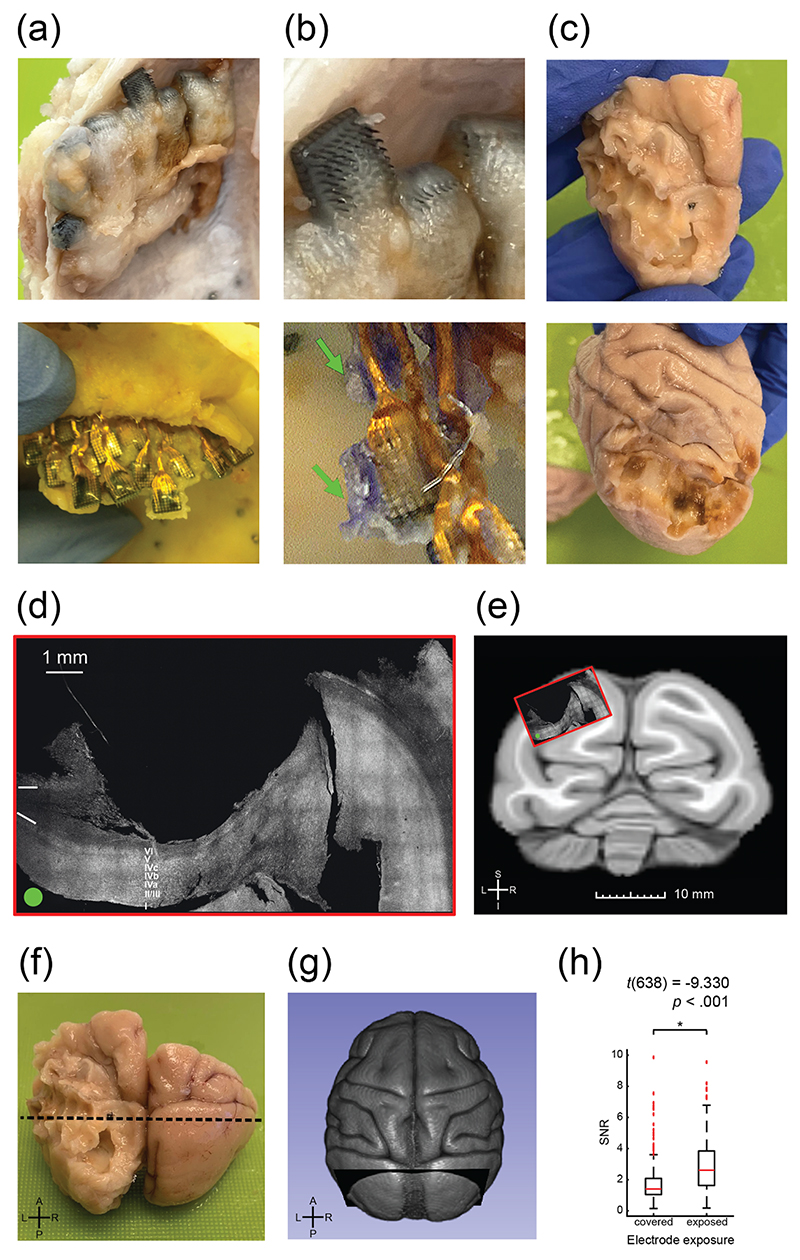
Tissue response and histology. (a), Photos of the brain and implant in monkey (a). Top: encapsulated arrays and wire bundles. Bottom: rows of arrays after explantation. (b), Top: close-up of partially encapsulated arrays. Bottom: tissue glue on explanted array and wire bundle, indicated by green arrows. (c), Photographs showing the surface of the visual cortex in monkeys L (top) and (a) (bottom). (d), Histological slice in monkey L, revealing lesioned cortex at implantation location, spanning the entire depth of dorsal V1 and underlying white matter. White lines demarcate white- and grey-matter boundaries. In the macaque brain, V1 cortex is folded, with part of V1 at the surface of the brain and the folded part forming a second layer underneath. The implant caused a large lesion in V1 cortex, revealing the underlying folded V1 cortex (layers of cortex are labelled). (e), Coronal slice from the co-registered NMT v2 template shown in G), corresponding to the dotted line in (f) and plane in (g). The overlay shows the approximate location of the histological slice from (d). S: superior; I: inferior; L: left; R: right. F, Fixed and extracted partial brain from monkey L (including occipital and parietal lobes), with visible damage to the implanted left hemisphere. Dotted line indicates approximate location of slice made during histology. A: anterior; P: posterior. G, 3D rendering of NMT v2 MRI template, registered to anatomical T1-weighted brain scan for monkey L before electrode array implantation. H, SNR obtained on electrodes with exposed versus encapsulated tips, combined across 10 arrays (5 per monkey). SNR was significantly higher on electrodes with exposed tips *t*(638) = −9.330, *p* < .001).

We dissected the tissue mass encapsulating the implants to reveal the arrays, electrodes, wire bundles, and reference wires. The relative positions of the arrays were similar to those during implantation (figure 6(a)). The histoacrylate tissue glue that had been applied to wire bundles and the sides of arrays during surgery to hold them in place was still clearly visible (figure 6(b), bottom).

### 3.8 Macroscopic and microscopic cortical damage

In both monkeys, the cortical surface of the implanted hemisphere was deformed, suggesting that it had been pushed downwards by the encapsulating tissue. Rows of depressions produced a scalloped appearance (figure 6(c)). Distinct depressions were identifiable for 9 of the 16 arrays in monkey L, ranging from 1.7 to 3.3 mm in depth, and for 8 arrays in monkey A, ranging from 1.9 to 5.3 mm in depth. The cortex of the non-implanted hemisphere appeared to be normal.

Macroscopic and microscopical analysis revealed that the cortex in monkey L was lesioned at the location of the electrode arrays. To visualize the extent of the lesion in monkey L, we overlaid the histological slice of the implanted region of cortex on a pre-surgical T1-weighted MRI scan, to which we co-registered a standard rhesus macaque template (methods) (figures 6(d)–(g)). The lesion spanned the entire depth of dorsal V1, as well as part of the underlying white matter (figures 6(d) and (e)). To correct for post-mortem shrinkage of the tissue, we scaled the histological section to match that of the pre-surgical scan. The extent of tissue damage can be seen in figure 6(e).

The cortical damage caused by the implants was in keeping with the loss of neuronal signals, the decreased number of phosphene-producing electrodes, and the poorer visual performance in the corresponding region of the visual field.

### 3.9 Electrode tip encapsulation

During tissue dissection, we found that newly formed tissue encapsulated the arrays and wires (figure 6(a)), flanking the upper and lower surfaces of the arrays and electrodes. The tissue mass was firmly attached to the dura but could be separated from it with tweezers. Five arrays in each of the monkeys (monkey L: arrays 4, 6, 8, 14, 15; monkey A: arrays 2, 6, 12, 14, 16) were only partially encapsulated, leaving several electrode tips exposed (figure 6(b), top).

As expected, electrodes with more tip encapsulation had lower SNR, an effect that was confirmed for seven of 10 arrays (*p*s < .01, Student’s unpaired *t*-test, Bonferonni correction, see supplementary table 1 for list of *p* values) and also when electrodes were pooled across monkeys and arrays. The SNRs of exposed and encapsulated electrodes were 3.1 ± 1.9 and 1.7 ± 1.0 (mean ± SD), respectively (figure 6(h), *t*(638) = *—* 9.330, *p* < .001, unpaired *t*-test). There was no significant difference in impedance between electrodes with exposed versus encapsulated tips (monkey L: *t*(318) = 1.9, *p =* .06; monkey A: *t*(318) = 1.0, *p* = .3, unpaired *t*-test). We also did not observe a difference in SNR between electrodes that had been stimulated and those that had not.

### 3.10 Scanning electron microscopy and electrode impedance

**Figure 7:**
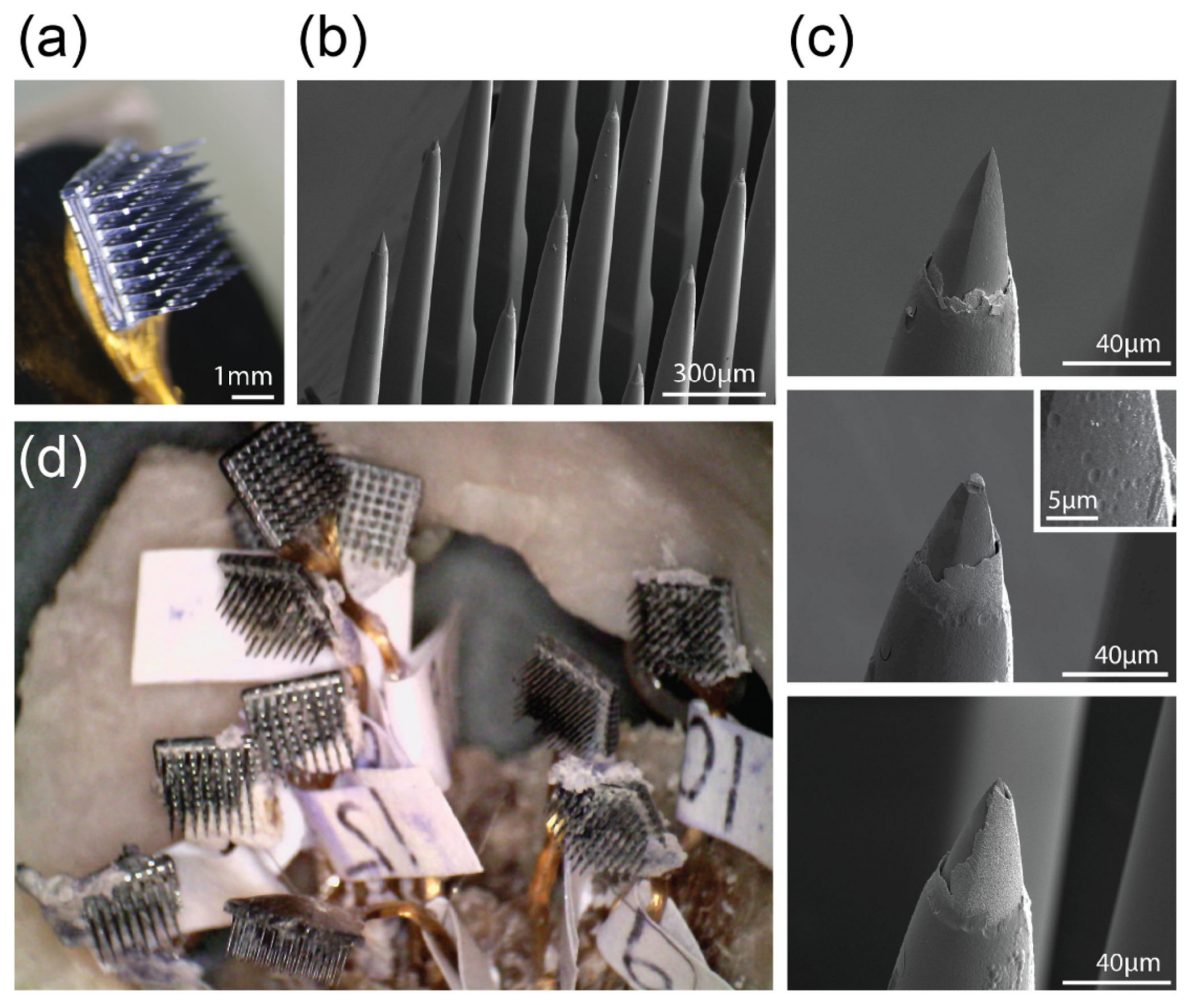
Explanted arrays. (a), Photograph of array 8, explanted from monkey L. (b), Close-up SEM image of array 8. (c), Scanning electron microscopy images of example electrodes on this array with missing or damaged IrO*x* layer, broken Si tip (middle), and pitting of Si surface (middle inset). (d), Photograph of arrays explanted from monkey (a).

We performed scanning electron microscopy on 62/64 electrodes from one array (array 8, figures 7(a)–(c)) in monkey L, and observed loss of the IrO*x* coating on 61/62 electrodes. Pitting of the exposed Si surface was observed on 13/62 electrodes (figure 7(c), inset). The Si tip itself remained intact in 5 electrodes and was damaged in the other 57 electrodes (figure 7(c), compare upper panel to middle and lower panel).

The *in-vivo* electrode impedances (measured 1299 d after surgery) were lower for electrodes with intact than with broken Si tips (*μ*_intact_: 479.5 ± 17.7 (SD) kOhms, *μ*_broken_: 503.0 ± 6.3, *t*(60) = − 2.93, *p* = .0047, unpaired *t*-test), implying that higher *in-vivo* impedances may indicate tip damage. There was no significant relationship between tip damage and SNR (*t*(60) = − 0.021, *p =* .983, unpaired *t*-test), and no effect of pitting on either SNR (*t*(60) = *—* 0.931, *p =* .356) or impedance (*t*(60) = −1.51, *p =* .137). There was also no relation between pitting and electrical stimulation (*χ*(2,62) = 0.166, *p =* .684, Chi-square test).

In monkey A, we examined *in-vivo* and *ex-vivo* impedances on the ten arrays that remained connected to the pedestal via their wire bundle (figure 7(d)). To our surprise, the impedance increased following explantation, from 45 ± 41 (mean ± SD) to 68 ± 84 kOhms (*t*(501) = − 5.4, *p* < .001, paired *t*-test), even though we removed connective tissue and cleaned the electrodes.

## 4 Discussion

Utah arrays have proven to be effective for both recording from motor cortex and stimulation of somatosensory cortex over many years in humans (>1500 d [54]), motivating us to consider their suitability for stimulation of the visual cortex with 1024-channel prosthesis. We monitored the efficacy of the device, neuronal signal quality, and the behaviour and general well-being of two monkeys over *~*3 years of implantation, as well as the tissue response.

The animals remained healthy throughout the implantation period, despite occasional signs of inflammation, which was kept under control by cleaning of the skin and the implant. However, across several years of implantation, the numbers of channels yielding reports of phosphenes during microstimulation decreased, and in one animal, the minimal amount of current necessary to elicit a phosphene increased. Furthermore, the SNR and the peak-to-peak voltage of the electrophysiological signals decreased over *~* 15 months in the first monkey and *~*4 months in the second monkey, matching the time course of the decline in the efficacy of phosphene generation. When we excluded the channels with high impedance values (>3000 kOhms), we observed decreases in the impedance of the remaining channels over the course of our study.

Post-mortem analyses revealed that the arrays and wire bundles were almost fully encapsulated, insulating them from the cortex after 3–3.5 years of implantation. The newly formed fibrotic tissue deformed the cortex, causing a visual impairment at the corresponding region of the visual field, in the form of decreases in accuracy and longer reaction times on a stimulus detection task.

Our analysis of electrode integrity after the experiment revealed degradation of the IrO*x* deposition layer, electrode tip breakage, and pitting of the electrode surface. Similarly, across three arrays in an nonhuman primate (NHP), Patel *et al* found that their arrays remained largely intact but individual electrodes showed signs of tip breakage and cracking, 590–848 d after implantation [65].

### 4.1 Implications for clinical translation

Silicon-based arrays such as the Utah array have been a staple of electrophysiology since the 1990s, allowing acute and chronic recording and stimulation of neuronal tissue in humans and NHPs. Such implants remain stable for months to years, allowing neuroscientists to observe and modulate brain activity within the same regions of tissue or groups of neurons for extended periods of time. These arrays have yielded significant breakthroughs in fundamental neuroscience and clinically applied technologies, allowing the generation of visual percepts in the blind [19] and enabling paralysed people to regain control over their muscles [66, 67], communicate [68], and control a computer cursor [69] or robotic arm [70–72] with their thoughts.

To date, only a small number of human patients have received intracortical Utah array implants (18 out of 48 patients having been chronically implanted for >30 d as of September 2018) [73] and the individual arrays contain 96 channels, which, in the case of a visual prosthesis, would provide limited phosphene coverage of visual space (~ 3 dva [19]). Hence, it remains unknown whether a high-channel-count device such as the 1024-channel implant that was used in monkeys [33] would be suitable for vision restoration in a clinical setting.

In NHPs, the longevity and efficacy of 96-channel Utah arrays varies substantially between subjects. Our results are consistent with previous studies demonstrating that signal quality decreases and tissue gliosis builds up over a period of months to years [62], a process that is accompanied by cortical atrophy, macrophage infiltration and neuronal necrosis [74]. These effects are thought to be caused by the presence of arrays rather than the delivery of stimulation because there are no histological differences between stimulated and non-stimulated tissue [74, 75], or behavioural differences in sensitivity to stimulation between heavily versus sparingly stimulated electrodes [76], and stimulation has even been reported to increase SNRs [77]. In our study, we did not observe a detrimental effect of stimulation either.

### 4.2 Causes of implant failure

The known causes of implant failure can be classified into three categories [62]. The first is material failure, e.g. degradation of insulating material on the probe or electrode tip breakage. The second is mechanical failure, e.g. damage to the wire bundle or connector, and the third is biological failure, caused by tissue trauma during initial electrode insertion and chronic immune system reactions to a foreign object. A survey of 78 silicon-based microelectrode arrays (including the Utah array) across 27 monkeys [62] showed that implant failure typically occurred within a year of implantation, due to mechanical issues such as problems with the connector. In the case of biological failures, progressive meningeal encapsulation of arrays was observed, separating the electrodes from the brain tissue. Encapsulation causes a gradual decline in spike amplitude over several years and damage to the electrodes causes decreases in their impedance. Furthermore, a recent study with a Utah array in a human patient [78] revealed proliferation of the meninges, fibrosis, lymphocytic infiltrates, astrogliosis, and foreign body reaction around the electrodes, combined with microhemorrhages, neuronal loss, and subcortical white matter necrosis. A second study on Utah arrays in humans found that longer periods of implantation were accompanied by greater array encapsulation [64]. However, IrO*x* delamination and cracking of the insulation layer did not prevent recording of neural signals. Our results are consistent with these previous findings, and we additionally documented deficits in visual acuity that accompanied the tissue damage.

In our study, signal quality declined gradually, indicating that material or biological reasons were the primary factors for the declining efficacy. Signal quality is particularly important for applications requiring neuronal read-out and decoding, such as motor control following paralysis [54, 79]. For applications involving stimulation, signal quality provides an indicator of electrode proximity to the neurons, but may be less critical for device functionality that depends on the efficacy of stimulation.

Numerous interacting factors are thought to affect *in-vivo* impedances, including electrode surface area, glial encapsulation, protein adsorption, stimulation waveforms, and the integrity of electrode insulation layers and electrode coating [61, 80]. Previous studies have documented an initial increase in electrode impedance during the first week of implantation [64, 81, 82], followed by a gradual decrease [23, 62, 75, 81, 82]. As our *in-vivo* impedance measurements began 1–3 months after implantation, we could not observe the initial increase seen in previous studies, but we did observe a subsequent decrease. We did not find a significant correlation between *in-vivo* SNR and *ex-vivo* electrode impedance, indicating that impedance may not be a reliable indicator of signal quality. Furthermore, we did not find an effect of degree of tip encapsulation on electrode impedance, implying that encapsulation does not substantially alter impedance values. We note, however, that we did not monitor encapsulation of the electrode tips *in vivo*, neither did we examine tissue adherence to the tips microscopically. On an explanted array that underwent scanning electron microscopy, we observed that electrodes with broken tips had higher *in-vivo* impedance values than those with intact tips, confirming that impedance is an indicator of electrode integrity.

The degree of electrode tip encapsulation predicted loss of SNR, in accordance with the hypothesis that the declines in SNR and microstimulation efficacy were caused by the growth of encapsulation tissue. Hence, the implant failure was caused by the tissue response rather than insufficient long-term stability of the device. The biological encapsulation of arrays and extrusion of electrodes from the cortex poses a challenge for long-term implantation and it is important to better understand their root causes and possible mitigation strategies to allow for safe and effective chronic use in the future.

The amount of array encapsulation and tissue damage that we observed seems to exceed that reported in previous studies using Utah arrays. We note that our implantation methods differ from the standard approaches used in previous studies (both clinical and pre-clinical) in five aspects, which may contribute to the differences observed: (1) the high density of the arrays, which were spaced closer together than in previous studies. (2) The use of tissue glue to secure the arrays to the cortex, which may have been toxic. (3) The reduction in relative motion between the arrays, as the tissue glue joined the arrays and wire bundles into a larger platform. (4) The tethering of arrays to the skull via wire bundles and the presence of micromotion between the stiff electrodes and softer brain tissue, which may have led to tissue scarring [83]. (5) Mechanical compression of the visual cortex by the 1024-channel device and encapsulating tissue.

We found that the tissue glue formed hard, jagged crystals that were still present upon explantation (figure 6(b), bottom). However, meningeal encapsulation of Utah arrays (such as that seen in figures 7(c) and (d) of Barrese *et al* [62]) has been widely reported in NHPs, making the application of tissue glue unlikely to be the sole reason for encapsulation. The volume of the implant and its rigidity is a likely cause of the newly formed scar tissue, which probably contributed to the cortical deformation and damage. In addition, tethering may have played a role: in clinical implantations of small numbers of Utah arrays, neurosurgeons typically take great care to minimize tension on the wire bundle during placement of each array and to provide strain relief between the skull and array. In our monkeys, the wire bundles ran directly from the craniotomy to the arrays with little strain relief, possibly inducing friction between the electrodes and the tissue, which may have contributed to the tissue encapsulation.

Future studies should aim to decrease the tissue response and encapsulation. A possible solution would be to position the arrays and wire bundles such that tension on the wire bundles is minimized. Furthermore, the use of novel probe designs and materials [84–88], such as thin-film polymers, could lead to a reduction in implant volume and provide a closer match in stiffness (Young’s modulus) between the probes and surrounding tissue. If these advances serve to temper immune system reactions and improve device biocompatibility, they may usher in the next generation of brain interfaces for functional vision restoration.

## Supplementary Material

Supplementary Material

## Figures and Tables

**Table 1:** Summary of datasets used. Number and type of data for each monkey and dataset, and time of data collection relative to array implantation (rounded to the nearest year).

Dataset	Number/type of data		State of the animal
	Monkey L	Monkey A	
Current thresholding	102 sessions over 2 years	69 sessions over 1 year	Performing a behavioural task with electrical stimulation
Visual detection task	12 sessions in year 3	19 sessions in year 2	Performing a behavioural task with visual stimulation
Visually evoked activity	21 sessions over 2 years	8 sessions over 1 year	Performing a behavioural task with visual stimulation
*In-vivo* impedance	19 sessions over 2 years	9 sessions over 1 year	Not performing a behavioural task
*Ex-vivo* impedance	NA	After 3 years	NA
Tissue dissection	After 3 years	After 3 years	NA
Histology	After 3 years	NA	NA
SEM	After 3 years	NA	NA

## Data Availability

The data cannot be made publicly available upon publication because the cost of preparing, depositing and hosting the data would be prohibitive within the terms of this research project. The data that support the findings of this study are available upon reasonable request from the authors.
