# Supplementary material for: Chronic stability of a neuroprosthesis comprising multiple adjacent Utah arrays in monkeys
Source: J Neural Eng. Author manuscript; Available in PMC 2024 Dec 8. (PMC7617000; doi:10.1088/1741-2552/ace07e)
Supplement: Supplementary Material [file EMS185244-supplement-Supplementary_Material.pdf]

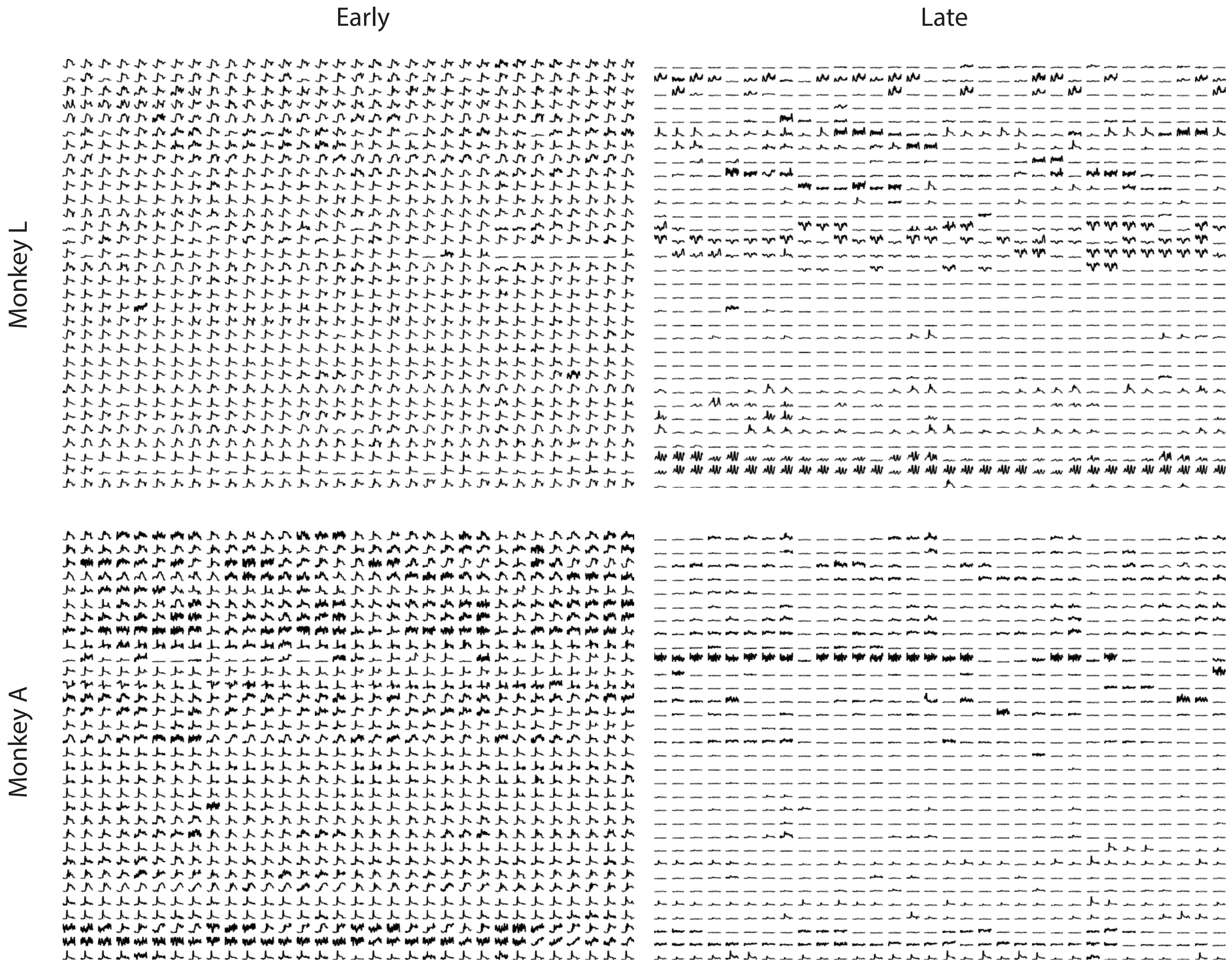

Supp Figure 1

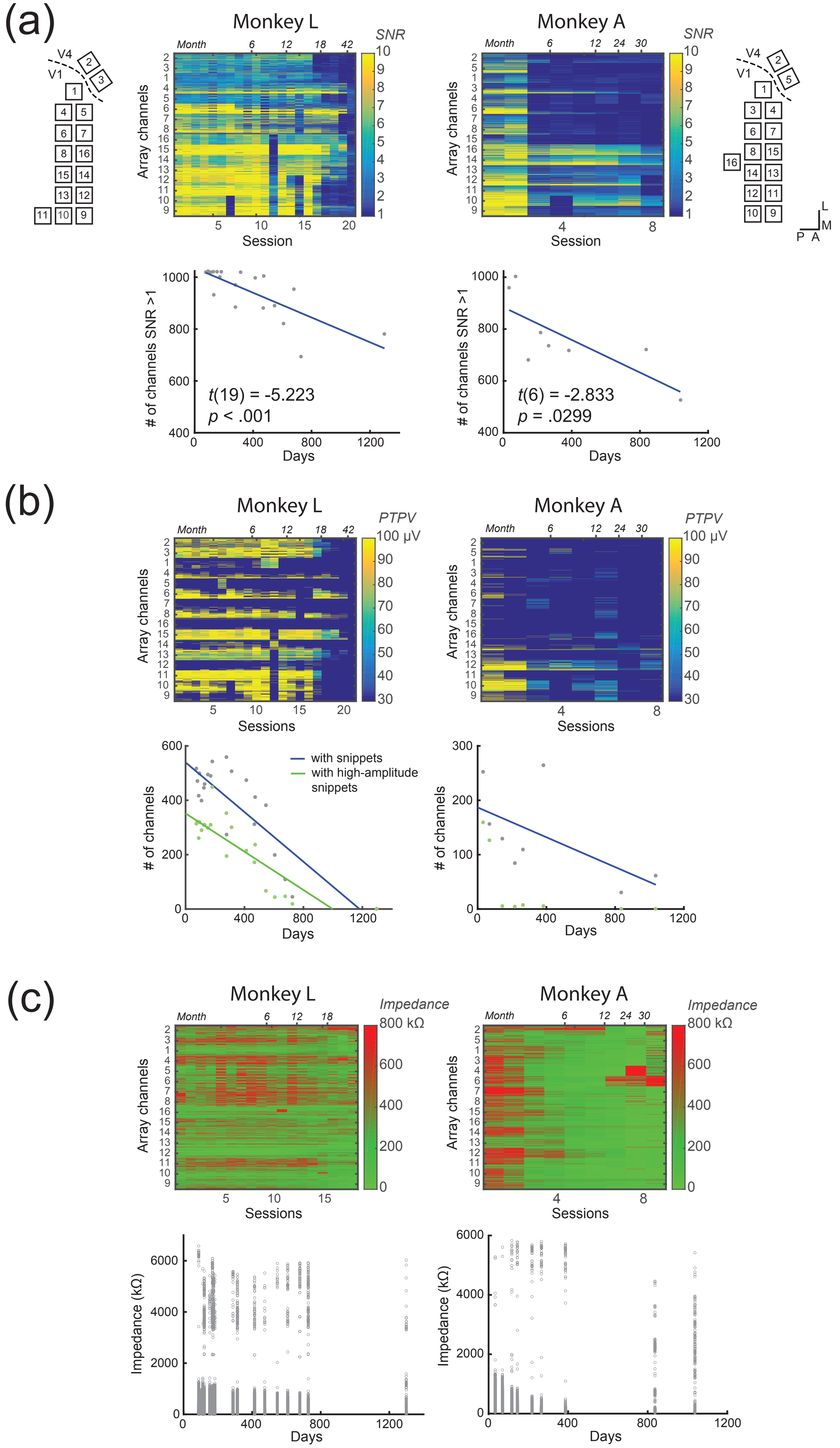

Supp Figure 2

Supplementary figure captions

Supplementary Figure 1. Activity elicited by a checkerboard stimulus across 1024 recording sites. Each curve shows the visually evoked response averaged across trials at every recording site during an example early (left) and late (right) session. For each channel, the range on the Y-axis is equal between early and late sessions. Across channels, the mean range of the Y-axis is 5.83 ± 5.79 (SD), in arbitrary units (a.u.).

Supplementary Figure 2. Changes in signal quality over time. A, top: Heat maps of SNR for 1024 channels, ordered by array. Each column in the heat map shows data from one session; time points are marked on the upper X-axis in 6-month intervals (note that sessions were not equally spaced across time). The schematic shows the approximate relative locations of arrays on the cortex and anterioposterior (AP) and mediolateral (ML) axes. Bottom: Number of channels with SNR values ≥1 across time. **B**, top: Heat maps of peak-to-peak voltage with time; time points are marked on the upper X-axis. Bottom: Number of channels with detectable (blue; snippets were detected if the signal exceeded 4.5 times the RMS level of spontaneous activity) and high-amplitude (>100 μV, green) snippets. **C**, top: Heat maps of impedance across time; time points are marked on the upper X-axis. Bottom: Impedance across time (measured at 1 kHz) for all channels, including high-impedance channels (>2000 kOhms), showing a bimodal distribution of impedance values across all the channels.

Supplementary tables

Monkey	Array number	*t*-statistic	*p* value
L	4	$t(62) = -3.188$	$p = .0022$
	6	$t(62) = 0.150$	$p = .881$
	8	$t(62) = 0.211$	$p = .833$
	14	$t(62) = -6.042$	$p < .001$
	15	$t(62) =-4.738$	$p < .001$
A	2	$t(62) = -3.6712$	$p < .001$
	6	$t(62) = 0.267$	$p = .790$
	12	$t(62) = -9.716$	$p < .001$
	14	$t(62) = -3.166$	$p = .0024$
	16	$t(62) = -6.279$	$p < .001$

Supplementary Table 1. Relation between SNRs and electrode tip exposure. In each monkey, we found 5 partially encapsulated arrays. Electrodes on these arrays were categorised as having either encapsulated or exposed tips. On 7/10 arrays, electrodes with exposed tips had significantly higher SNRs than those with encapsulated tips.
